# Elucidating the pressure-induced enhancement of ionic conductivity in sodium *closo*-hydroborate electrolytes for all-solid-state batteries

**DOI:** 10.1007/s10853-022-08121-8

**Published:** 2023-01-14

**Authors:** Yuanye Huang, Radovan Černý, Corsin Battaglia, Arndt Remhof

**Affiliations:** 1 Empa, Swiss Federal Laboratories of Materials Science and Technology, Dübendorf, Switzerland; 2 DQMP, University of Geneva, Quai Ernest-Ansermet 24, 1211 Geneva, Switzerland

## Abstract

Hydroborates are an emerging class of solid electrolytes for all-solid-state batteries. Here, we investigate the impact of pressure on the crystal structure and ionic conductivity of a close-hydroborate salt consisting of Na_2_B_10_H_10_ and Na_2_B_12_H_12_. Two Na_2_B_10_H_10_:Na_2_B_12_H_12_ ratios were studied, 1:1 and 1:3. The anions of the as-synthesized powder with 1:1 ratio crystallize in a single face-centered cubic phase, while the anions of the powder with 1:3 ratio crystallize in a single monoclinic phase. After applying pressure to densify the powder into a pellet, a partial phase transformation into a body-centered cubic (BCC) phase is observed for both ratios. The BCC content saturates at 50 weight percent (wt%) at 500 MPa for the 1:1 ratio and at 77 wt% at 1000 MPa for the 1:3 sample. The room temperature sodium-ion conductivity follows an analogous trend. For the 1:1 ratio, it increases from 2 × 10^–4^ Scm^−1^ at 10 wt% BCC content to about 1.0 × 10^–3^ Scm^−1^ at 50 wt% BCC content. For the 1:3 ratio, it increases from 1.3 × 10^–5^ Scm^−1^ at 11.9 wt% BCC to 8.1 × 10^–4^ Scm^−1^ at 71 wt% BCC content. Our results show that pressure is a prerequisite to achieve high sodium-ion conductivity by formation of the highly conductive BCC phase.

**Supplementary Information:**

The online version contains supplementary material available at 10.1007/s10853-022-08121-8.

## Introduction

Alkali metal hydroborates are receiving increasing attention as electrolytes for all-solid-state batteries. Especially the lithium and sodium *closo*-borate salts with *closo*-caged B_n_H_n_^2−^ or CB_n-1_H_n_^−^ anions combine favorable material properties such as compatibility with lithium and sodium metal anodes, high oxidative stability (> 3 V vs. Li/Li^+^ and Na/Na^+^), low gravimetric densities (≤ 1.2 g cm^−3^); high thermal and chemical stability, soft mechanical properties enabling cold pressing, solution processability and low toxicity [1, 2]. Hydroborates typically undergo order/disorder phase transitions from a phase with low cation conductivity to a phase with high cation conductivity at elevated temperatures. Prominent examples are Na_2_B_10_H_10_ and Na_2_B_12_H_12_ *closo*-borates that transform from a low symmetry room temperature monoclinic phase to face-centered cubic (FCC) and body-centered cubic (BCC) phases at temperatures of 100 °C [3] and 260 °C [4], respectively. The high-temperature FCC phase can be stabilized at room temperature when mixing Na_2_B_10_H_10_ and Na_2_B_12_H_12_ in an equimolar 1:1 ratio [5].

High symmetry of mixed-anion *closo*-hydroborate B_n_H_n_^2−^ and/or *closo*-hydromonocarbaborate CB_n-1_H_n_^−^ phases enables room temperature ionic conductivities above 10^–3^ S cm^−1^ [5, 6].

Stable cycling of a number of 3 V and 4 V class all-solid-state batteries based on such mixed-anion electrolytes was demonstrated previously [7, 8]. Typically, a pressure on the order of 300 MPa is applied to form a dense hydroborate separator and the hydroborate/cathode composite.

Even though pressure-induced phase transitions were reported previously for a number of hydroborates [9], no detailed study has yet considered the effect of pressure on phase content and the ionic conductivity of hydroborate pellets.

In the present study, combining X-ray diffraction and electrochemical impedance spectroscopy, we show that already relatively low pressure causes persistent segregation of a BCC phase from the 1:1 FCC and 1:3 monoclinic Na_2_B_10_H_10_:Na_2_B_12_H_12_ mixtures and results in a substantial enhancement of the ionic conductivity.

## Experimental

### Sample preparation

All samples were handled and stored in argon atmosphere (glovebox MBraun, H_2_ and O_2_ level < 0.1 ppm) or under vacuum.

**Figure 1:**
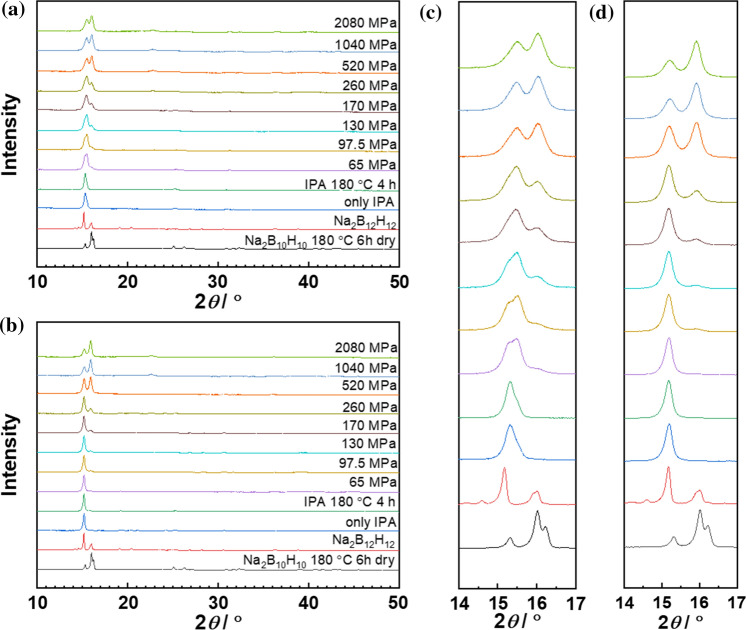
Normalized XRD patterns of the 1:1 sample (**a**, **c**) and of the 1:3 sample (**b**, **d**) under different pressure compared to raw materials and as prepared powders. **a**, **b** Display the patterns in the angular range between 10° and 50°, while **c**, **d** are the enlargement for 14°–17°. The same color-coding and order is used

Na_2_B_10_H_10_ (Katchem) was dried under vacuum (1.0 × 10^–3^ mbar) at 180 °C for 6 h using Schlenk techniques (denoted as 'Na_2_B_10_H_10_, 180 C, 6 h dry' in Fig. 1). Na_2_B_12_H_12_ (Katchem) was used as received (denoted as 'Na_2_B_12_H_12_' in Fig. 1). Stoichiometric amounts of pre-dried Na_2_B_10_H_10_ and Na_2_B_12_H_12_ (for 1:1 and 1:3 samples, respectively) in total of 1 g were dissolved in 20 mL isopropanol (IPA) in an ultrasonic bath. Subsequently, the solution was dried using a rotary evaporator under vacuum at 25 mbar in a 60 °C deionized water bath. Afterward, the flask was transferred to a Schlenk line under vacuum to be further dried at 100 °C until the vacuum value reached 1.0 × 10^–3^ mbar. Finally, a heat treatment at 180 °C for 4 h was applied, after which the powder was ground in a mortar for 15 min for the next step.

Pellets of 30 mg each were prepared by uniaxial cold pressing using a hardened steel die with a diameter of 6.35 mm. The die was sealed in a gas tight bag in the glovebox and transferred to a hydraulic press (Specac) where pressures between 65 and 2080 MPa were applied for 3 min. Subsequently the die was transferred back to the glovebox. All pellets reach are compressed to densities above 80% of the single crystalline density.

### Sample characterizations

Samples were sealed into borosilicate capillaries (diameter 0.7 mm) under inert conditions. The as-prepared powders were directly filled into the capillaries after grinding. The freshly pressed pellets and pellets after electrochemical impedance spectroscopy (EIS) measurement were first pulverized in a mortar and then filled into capillaries. The XRD patterns were collected on an XRD diffractometer (MalvernPanalytical Empyrean) operated at an accelerating voltage of 45 kV and a current of 40 mA. A focusing mirror in combination with Cu K_α1,2_ radiation was employed to focus the incident radiation onto the sample. The diffraction patterns were recorded using an X'Celerator detector in the angular range between 10° and 50° with a step size of 0.004°. Rietveld refinements of the patterns were performed using TOPAS [10]. The atomic coordinates were fixed at values published in literature and only cell parameters, peak shapes and scale factors were refined.

Electrochemical impedance spectroscopy (EIS) measurements were taken on a Novocontrol impedance analyzer. Pellets were sandwiched between two indium foils with a diameter of 6 mm for better electronic contact in an air-tight sample holder. The temperature of the sample was controlled via the flow of liquid nitrogen. The sample experienced two heating/cooling cycles. The first cycle was from − 20 to 100 °C, and the second one was from − 20 to 120 °C. For each temperature, the EIS measurement started after the temperature equilibrated at the set temperature ± 0.5 °C for at least 1 min. The impedance was measured in the range of 20 MHz to 1 Hz (for fitting only data below 1 MHz were used) with a 10 mV AC voltage. Because of the high conductivity, the bulk semicircle of the electrolyte was not visible and the spectra were fitted with a resistance (R) element in series with a constant phase element (CPE) element for the high-frequency linear part (see Figure S1).

## Results and discussion

### Pressure-induced phase transformation

Figure 1 shows the XRD patterns of the precursors, i.e., room temperature monoclinic Na_2_B_10_H_10_ (monoclinic *rt*-Na_2_B_10_H_10_ [11]), obtained after the drying process at 180 °C and monoclinic Na_2_B_12_H_12_ (monoclinic *rt*-Na_2_B_12_H_12_ [12]). Figure 1 further depicts the XRD pattern of the as prepared mixed-anion Na_2_B_10_H_10_:Na_2_B_12_H_12_ powders (recrystallized from IPA solution without and with post-crystallization heat treatment [13], and the corresponding samples after compaction into pellets at increasing pressure and subsequent pulverization. After dissolution in IPA and recrystallization, both 1:1 and 1:3 samples crystallized as a single phase. While the 1:3 sample maintains the structure of monoclinic *rt*-Na_2_B_12_H_12_, the 1:1 sample crystallizes with FCC symmetry. Under pressure, the main reflection of the 1:1 sample slightly shifts to higher angle and both compositions show peak splitting in the angular range between 14° and 17°, indicating a phase segregation. For the 1:1 sample, three phases can be identified at low pressure (65–170 MPa), indicated by overlapping reflections centered on 15.2°, 15.5° and 16.0°. The symmetry of these phases was identified as monoclinic, FCC and BCC, respectively. Due to the slight asymmetry of the leading reflection of the 1:1 sample toward larger angles, we cannot exclude the presence of the BCC already in the as-prepared state. The reflection at ~ 15.2° disappears at pressures above 200 MPa, signifying transformation of the monoclinic phase into FCC or BCC phase (Fig. 1a, c). For the 1:3 sample, the phase segregation shows a different behavior. Only two reflections at 15.2° and 16.0°, indicative of a monoclinic and a BCC symmetry, can be observed (Fig. 1b, d). For both ratios, the low-angle reflection dominates at low pressure. With increasing pressure, the reflection at ~ 16.0° (BCC) prevails more and more, and the intensity ratio of the reflection at 15.5° (for 1:1) or 15.2° (for 1:3) to the one at 16.0° saturates at 520 and 1040 MPa, respectively.

Rietveld refinement was applied to quantify the phase content. The published structures of the monoclinic *rt*-Na_2_B_10_H_10_ [11] and monoclinic *rt-*Na_2_B_12_H_12_ [12] were used as structural models for the pure phases. For the mixed-anion phases, the high temperature structures of Na_2_B_12_H_12_ were chosen as structural models for the FCC (${\text{Fm}}\bar{3}$) and BCC (${\text{Im}}\bar{3}m$) phases [14, 15]. This choice is justified by the anion orientation disorder presumed in the phases leading to practically zero diffraction contrast between B_10_H_10_^2−^ and B_12_H_12_^2−^ anions. In the present study, the as-prepared (non-pressed) 1:1 and the 1:3 mixtures crystallized as single phases contrary to the study of Yoshida et al., who observed the coexistence of the BCC phase together with the FCC phase (1:1) or monoclinic *rt*-Na_2_B_12_H_12_ phase (1:3), respectively, already in as-prepared samples [16]. We attribute this to different sample preparation methods, crystallization from a solution in our case and ball milling in Ref. [16]. As discussed by Yoshida et al., the difference in formation energy between the different phases are rather small; a 0.05 eV per Na_2_B_10_H_10_/Na_2_B_12_H_12_ difference between the 1:3 mixture and the pure phases was reported. Therefore, the sample preparation by ball milling as performed by Yoshida et al. may be sufficient to establish to BCC phase in the as-prepared samples. In fact, a ball milling-induced transformation to a BCC phase has been observed in NaCB_11_H_12_ [17].

**Figure 2:**
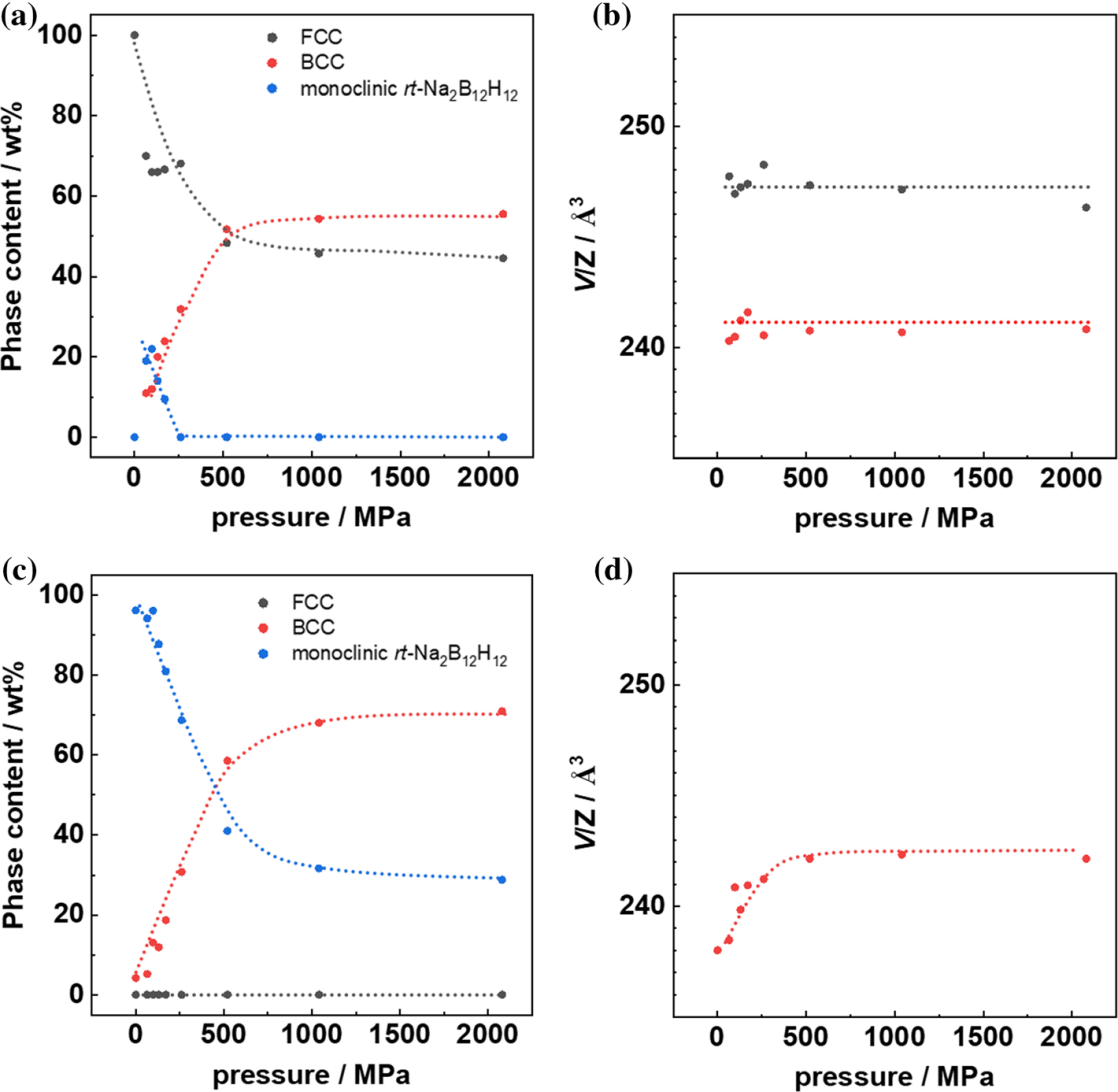
**a**, **c** phase contents, **b**, **d** cell volume per unit formula (*V*/Z) of BCC (red) and FCC (black) phases versus pressure. **a**, **b** Are from the 1:1 sample and **c**, **d** are from the 1:3 sample. The dashed lines are only a guide for the eye

As shown in Fig. 2a, c, at low pressure of 65 MPa, the 1:1 sample segregates into three different phases, i.e., a FCC, a BCC and a monoclinic phase, whereas the 1:3 sample develops a BCC phase next to the initially present monoclinic phase. For the 1:1 sample, both FCC and monoclinic phase contents decrease with increasing pressure, and the monoclinic phase disappears at ~ 300 MPa. The BCC phase content, on the other hand, increases on the expense of the FCC and the monoclinic phases. At ~ 500 MPa, the FCC and the BCC phase contents balances at a ratio comparable to the nominal ratio of Na_2_B_10_H_10_:Na_2_B_12_H_12_ (M(Na_2_B_10_H_10_):M(Na_2_B_12_H_12_) = 164.16 gmol^−1^:187.796 gmol^−1^). Throughout the pressure range used for sample preparation between 65 and 2080 MPa, the unit cell volumes of the FCC and BCC phases remain constant at 483 Å^3^ for the BCC and 988 Å^3^ for the FCC phase, corresponding to 241 Å^3^ and 247 Å^3^ per Na_2_B_x_H_x_ formula unit. The surprisingly smaller volume per formula unit in the BCC phase as compared to the FCC phase has recently been observed in chemically related NaCB_11_H_12_, a detailed discussion can be found in Ref. [17]. The authors argue that the reason for this unusual behavior is the optimized anion packing within the BCC phase allowing closer anion–anion distances due to optimized hydrogen–hydrogen contacts. This optimized packing is proposed to be one of the driving forces toward the BCC symmetry. In addition, the enhanced number of available sodium sites in the BCC lattice with respect to the FCC lattice contributes to the configurational entropy, further stabilizing the BCC phase and increasing the ionic conductivity, as discussed below. Due to the small amount of the monoclinic phase and the resulting low-intensity reflections, its lattice constant was fixed to the value of the pure Na_2_B_12_H_12_ phase during refinement.

Different from the 1:1 sample, the as-prepared 1:3 sample crystallizes in a monoclinic phase, with a unit cell volume of 511 Å^3^, which roughly matches the weighted average of the unit cell volumes of the monoclinic phases of Na_2_B_10_H_10_ and Na_2_B_12_H_12_. As in the 1:1 case, with increasing pressure, the BCC phase develops and saturates at about 1000 MPa at 71 weight percent (wt%), slightly below the initial Na_2_B_12_H_12_ content of 77.4 wt%. It assumes a unit cell volume of 485 Å^3^ (242.5 Å^3^ per formula unit) which is comparable to the unit cell volume of the BCC phase in the 1:1 case. Within the 1:3 case, a slight increase of the unit cell volume with pressure is observed at pressures below 500 MPa which is small compared to the difference in unit cell volume of the individual Na_2_B_10_H_10_ and Na_2_B_12_H_12_ phases. From the comparison with the unit cell volume of the BCC NaCB_11_H_12_ of 244.8 Å^3^, we can conclude that in our case, the BCC phase contains a mixture of B_10_H_10_^2−^ and B_12_H_12_^2−^ anions. It indicates that the phase separation is more a partial structural transformation than a separation into phases of different stoichiometries and can be explained by a displacive transformation, which is of martensitic (lattice distortion) type in our case [18]. The transition between FCC and BCC symmetry is not uncommon in nature. This diffusionless, martensitic transition is well known as a temperature-induced phase transition in steel [18, 19]. Generally, we observe the same trend as Yoshida et al. [16], B12-rich phases tend to favor the BCC phase while B10-rich samples favor the FCC phase. The 1:3 ratio was chosen as it was reported to maximize the amount of the BCC phase.

### Persistent enhancement of ionic conductivity

**Figure 3:**
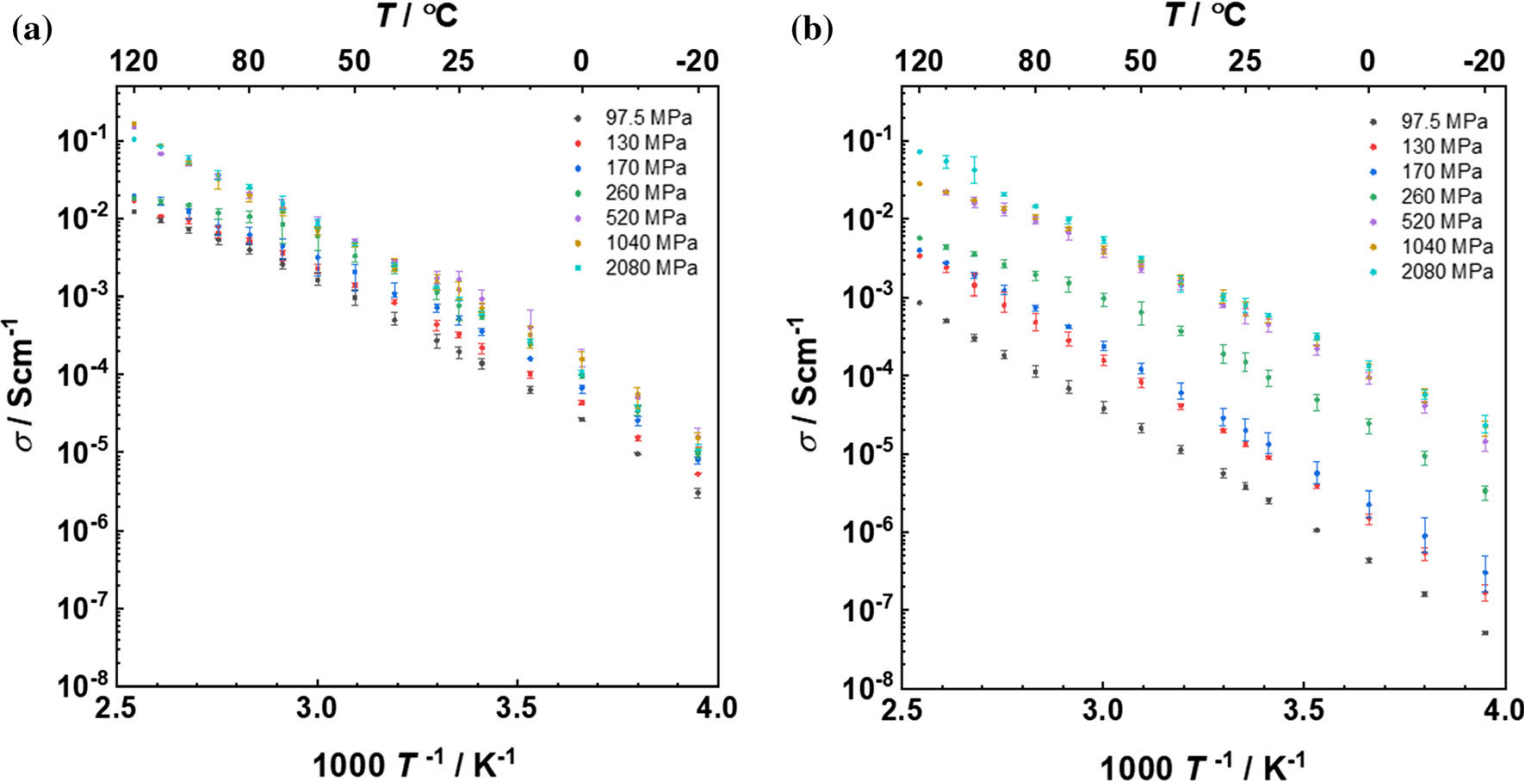
Arrhenius plots of **a** 1:1 and **b** 1:3 samples under different pressure. Two heating and cooling cycles were applied. Average value and the absolute error bars (both plus and minus) were calculated for each temperature

EIS measurement reveals a clear correlation between the ionic conductivity and the applied pressure during pellet preparation. As shown in Fig. 3, both 1:1 and 1:3 samples exhibit an increasing ionic conductivity with pressure, an effect that is more pronounced for the 1:3 samples. From 97.5 to 2080 MPa, the room temperature ionic conductivity of the 1:1 sample increases from 2 × 10^–4^ S cm^−1^ to about 1 × 10^–3^ S cm^−1^, while one of the 1:3 samples increases from 1.3 × 10^–5^ to 8.1 × 10^–4^ S cm^−1^. In the 1:1 sample (Fig. 3a), a constant activation energy of 0.6 eV in the temperature range of – 20 to 70 °C is observed, regardless of the previously applied pressure. At higher temperature from 70 to 120 °C, the activation energies for the samples pressed at lower pressure (< 520 MPa) decrease to about 0.35 eV, while the one for the samples pressed at higher pressure (i.e., $\ge$ 520 MPa) remains constant. A change in apparent activation energy from 0.60 to 0.35 eV has been reported by Duchene et al. in a 1:1 sample prepared by ball milling and pressed at 300 MPa [20] and was also observed in other anionic mixtures [6] and in BCC NaCB_11_H_12_ [17]. Duchêne et al. noticed that the apparent activation energy of 0.6 eV at room temperature is higher than expected from the local microscopic barrier of 0.35 eV observed by, e.g., ^23^Na nuclear magnetic resonance spin–lattice relaxation. This behavior was attributed to the coupling of the cation and anion motion due to short-range ion–ion interactions combined with background energy fluctuations, leading to correlated ion diffusion. Thereby, the background energy fluctuations could be associated with fast liberations of the anions. In the temperature regime above 70 °C, the thermal energy becomes sufficiently high so that these energy fluctuations become less relevant and thus sodium diffusion then occurs with a lower activation energy of the uncorrelated motion [20]. Obviously, in the BCC phase that dominates at higher pressures, the correlations between the anions and the cations are more pronounced and temperatures between 70 and 120 °C are not sufficient to uncouple their motions.

For the 1:3 sample, which initially crystallizes in a different symmetry than the 1:3 samples, a similar behavior can be observed. Pellets pressed at intermediate pressure, i.e., 260–1040 MPa, also show two regimes. Same as the 1:1 sample, 1:3 pellets have a comparable apparent activation energy of 0.6 eV at temperatures between − 20 and 70 °C. As for the 1:1 samples, the apparent activation energy changes to 0.35 eV at higher temperatures and we assume a similar conductivity mechanism. Exceptions are the samples pressed at 97.5 and 2080 MPa, which do not undergo a change in activation energy. Currently, this behavior requires further studies to unveil their conduction mechanism. In Fig. 3, we plot *σ* versus (1/*T*) for clarity. The activation energy is extracted from the slop of (*σT*) versus (1/*T*). A current constriction effect for low-pressure samples can be ruled out, as (1) all measured samples have a relative density higher than 80% and (2) the activation energies at low temperature (− 20 to 70 °C) are comparable with the ones determined for high-pressure samples in the range of 0.6 eV.

For all the samples, no change was observed after two heating/cooling cycles and EIS measurements from the XRD patterns (see Figure S2). It shows that the phase segregation behavior is thermally stable in the measurement range of − 20 to 120 °C.

**Figure 4:**
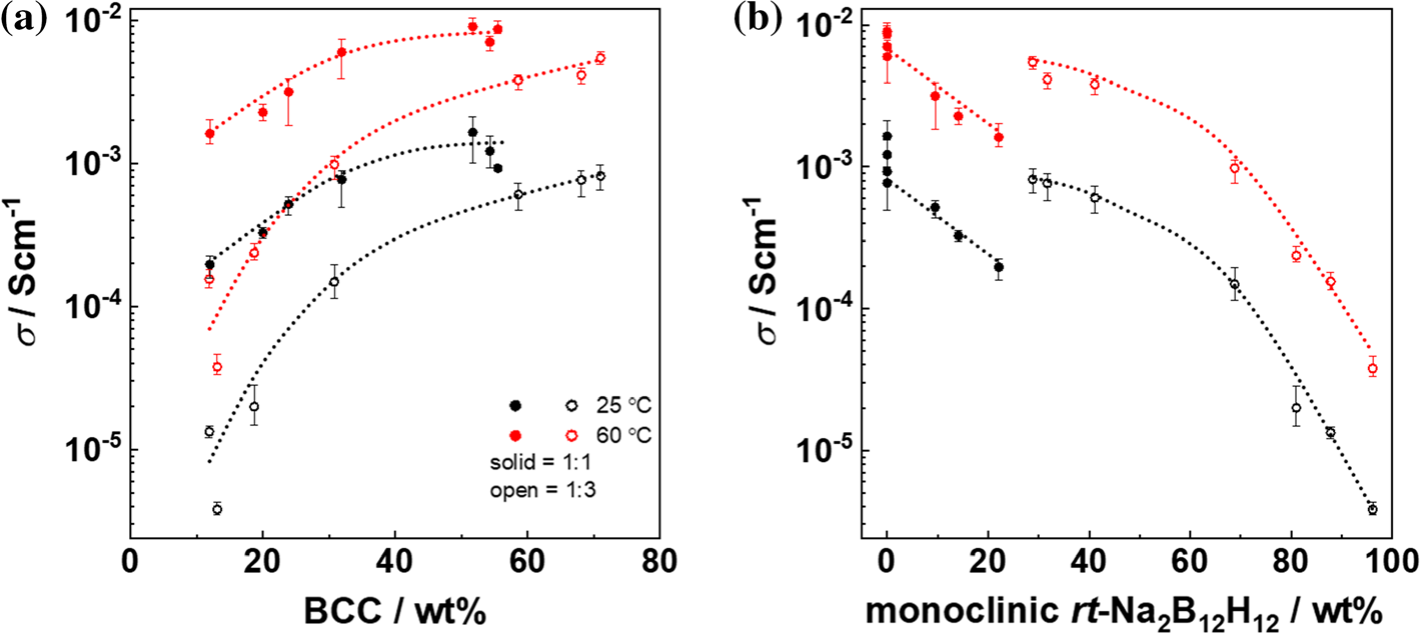
Correlation of conductivity at 25 °C and 60 °C with phase content **a** versus the BCC phase and **b** versus the monoclinic *rt*-Na_2_B_12_H_12_ phase. The dashed lines are only a guide for the eye

Another way of looking at the conductivity is its dependence of the phase composition. In Fig. 4, we plot the ionic conductivity against BCC (Fig. 4a) and monoclinic (Fig. 4b) phase contents, respectively. Values at both 25 and 60 °C are shown as typical operation temperatures for hydroborate batteries. Though the ionic conductivity is typically higher at elevated temperature, the curves show the same trend.

As shown in Fig. 4a, the BCC phase is clearly beneficial for the ionic conductivity. Especially for the 1:3 sample, at 25 °C, with 11.9 wt% BCC phase the conductivity is as low as 1.3 × 10^–5^ Scm^−1^ and increases up to 8.1 × 10^–4^ Scm^−1^ at a BCC content of 71 wt%, while it increases from 1.6 × 10^–4^ Scm^−1^ to 5.4 × 10^–3^ Scm^−1^ at 60 °C. For the 1:1 sample, the increase in the conductivity with BCC phase content is less pronounced. The conductivity increases from 2.0 × 10^–4^ Scm^−1^ to ~ 1.0 × 10^–3^ Scm^−1^ at 25 °C, and 1.6 × 10^–3^ Scm^−1^ to ~ 9.0 × 10^–2^ Scm^−1^ at 60 °C as the BCC contend increases from 12 to 55.5 wt%.

The different level of impact of BCC phase content on the ionic conductivity can be related to the monoclinic phase, which is detrimental as shown in Fig. 4b. Notable, from 28.8 to 96.1 wt% monoclinic *rt*-Na_2_B_12_H_12_ phase, the conductivity decreases over 2 orders of magnitude. The detrimental effect of the monoclinic phase overcomes over the beneficial effect of the BCC phase, and therefore, even with the same BCC content, the 1:3 sample with a higher monoclinic phase contend has a lower conductivity than the 1:1 sample. On the other hand, the 1:3 sample has a higher BCC phase content at higher pressure (cf. Fig. 2a, d) and reaches comparable conductivity value as the 1:1 sample.

We attribute therefore the increase of ionic conductivity with applied pressure is the partial transformation to the BCC symmetry. In general, BCC symmetry possesses the lowest diffusion barriers and enables therefore higher ionic conductivities compared to other crystal symmetries. Within the BCC symmetry, ions can diffuse along a path connecting two face-sharing tetrahedral sites [21]. Another parameter beneficial for the ionic conductivity is three times higher number of tetrahedral sites per anion in BCC compared to FCC [21], which correspondingly increase the entropy parameter in the pre-exponential term of Arrhenius equation [17].

## Conclusion

In summary, we have shown that mixed Na_2_B_10_H_10_:Na_2_B_12_H_12_ in a 1:1 and 1:3 ratio can be prepared from solution as phase pure compounds, crystallizing in a FCC and a monoclinic structure, respectively. Applying pressures that are typically used for the densification of the electrolyte or during battery cell assembly are sufficient to induce partial transformation into a BCC phase. Within the 1:1 mixture, the BCC contend saturates at 50% at pressures about 500 MPa, while the BCC content of the 1:3 phase reaches 71% at 1000 MPa. The ionic conductivities increase with increasing BCC content, reaching 1.0 × 10^–3^ Scm^−1^ and 8.1 × 10^–4^ Scm^−1^ at room temperature, respectively. The transformation persists pressure release, is stable at elevated temperatures and withstands the application as a solid electrolyte in a battery. It is the cause for the high ionic conductivities in these compounds; its robustness is the prerequisite for the application as a solid electrolyte. Na_2_B_10_H_10_:Na_2_B_12_H_12_ is not unique in this respect. Hydroborates, as other plastic crystals, often display complex phase diagrams and polymorphs with similar formation enthalpies. Ball milling is a known technique to use their structural flexibility to tune physicochemical properties, especially the ionic conductivity. This study demonstrates that also the application of pressure should be considered to increase and to understand the ionic conductivity in anion mixed hydroborates.

Considering the cost of the building blocks, Na_2_B_10_H_10_ is three times more expensive than Na_2_B_12_H_12_. By partially substituting Na_2_B_10_H_10_ by Na_2_B_12_H_12_ and applying suitable pressure, the cost for the solid electrolyte may decrease about 25% while maintaining comparable conductivity. The result is promising, although further work on integrating the 1:3 electrolyte into an all-solid-state battery is needed.

## Supplementary Information

Below is the link to the electronic supplementary material.

Supplementary file1 (The supplementary information contains (i) an exemplary EIS spectrum measured at 25 °C from 1:1 sample pressed at 2080 MPa and (ii) XRD patterns after EIS measurement for (a) 1:1 and (b) 1:3 samples)

## Data Availability

Not applicable.
