# Supplementary material for: Elucidating the pressure-induced enhancement of ionic conductivity in sodium closo-hydroborate electrolytes for all-solid-state batteries
Source: J Mater Sci. 2023 Jan 14;58(17):7398–406. doi: 10.1007/s10853-022-08121-8 (PMC10160155; doi:10.1007/s10853-022-08121-8)
Supplement: Supplementary file 1 — Supplementary file1 (The supplementary information contains (i) an exemplary EIS spectrum measured at 25 °C from 1:1 sample pressed at 2080 MPa and (ii) XRD patterns after EIS measurement for (a) 1:1 and (b) 1:3 samples) [file 10853_2022_8121_MOESM1_ESM.docx]

**Supplementary information**:

**Elucidating the pressure induced enhancement of ionic conductivity in sodium *closo*-hydroborate electrolytes for all-solid-state batteries**

*Yuanye Huang^1^, Radovan Cerny^2^, Corsin Battaglia^1^, Arndt Remhof^1^*

^1^Empa, Swiss Federal Laboratories of Materials Science and Technology, Switzerland

^2^DQMP, University of Geneva, Quai Ernest-Ansermet 24, 1211, Geneva, Switzerland





Figure S 1: Exemplary EIS spectrum measured at 25 °C from 1:1 sample pressed at 2080 MPa. The equivalent circuit, which is composed of a resistance in series with a CPE element, is indicated in the graph. Here only the linear part of the spectrum below 1 MHz was used for the fitting. Due to the high conductivity, no semicircle from the electrolyte is observable and the interception of the linear fit with the real axis in the Nyquist plot is the resistance from the electrolyte R_electrolyte_ (Here the resistance from the instrument is considered to be negligible).


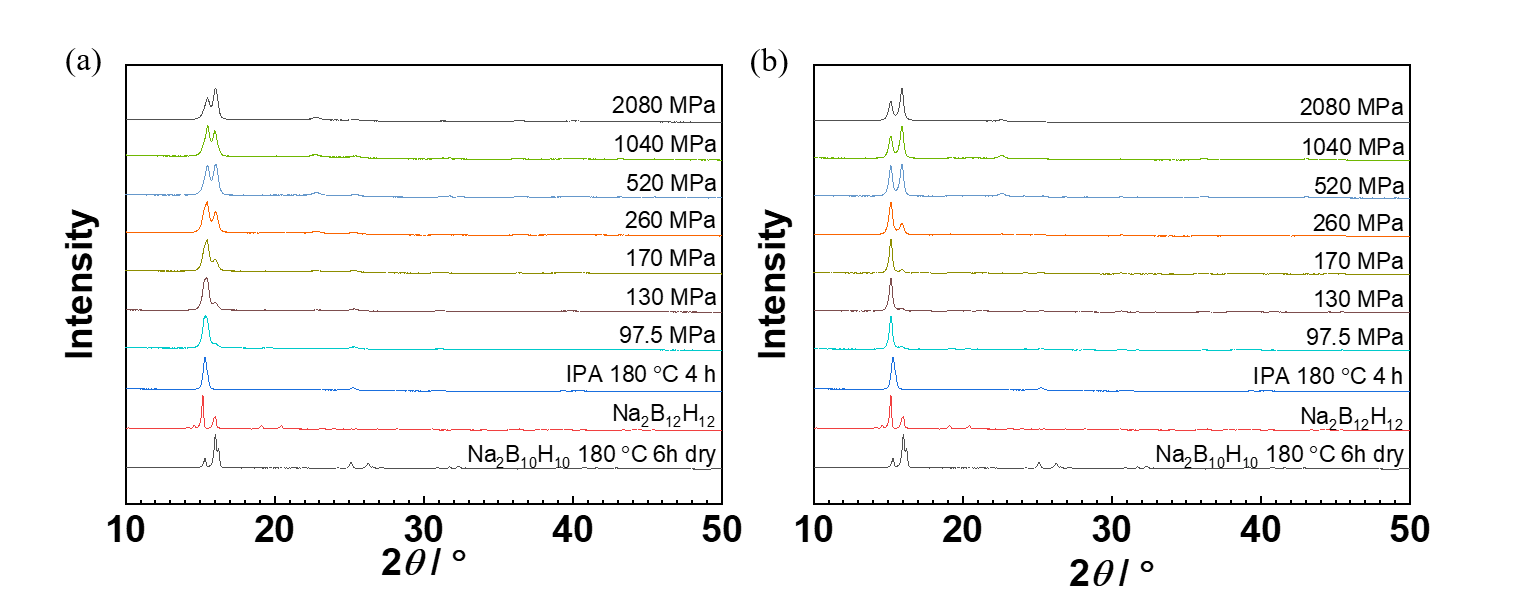


Figure S 2: XRD patterns after EIS measurement for (a) 1:1 and (b) 1:3 samples. The contacting electrodes used were In foils.
